# Right-Sided Pleural Effusion in a Critically Ill Stroke Patient

**DOI:** 10.1177/2324709614523258

**Published:** 2014-02-07

**Authors:** Alexander Bautista, Michael Heine, Victor van Berkel, Lydia Kelly-Frasher, Kerri Remmel, Ozan Akca

**Affiliations:** 1 University of Louisville, Louisville, KY, USA

**Keywords:** esophageal perforation, pleural effusion, transesophageal echocardiography, non–small cell lung carcinoma, critical care

## Abstract

Pleural fluid collections are common in those critically ill. We report the case of a left middle cerebral artery stroke patient who developed respiratory distress and required intubation and mechanical ventilation. Although the patient’s clinical status and oxygenation improved, there was persistence of right-sided opacity in the chest radiograph. Further workup proved a right-sided pleural effusion, which was drained and managed. Following extubation, a swallow study was ordered, which led to a fluoroscopic examination that demonstrated esophageal perforation. Thoracic surgery was consulted and did a primary repair of perforation and noted non–small cell carcinoma on the perforated site.

## Introduction

Pleural fluid collections are common in critically ill patients. Predominantly, these fluid collections are transudates and do not require drainage unless it is compromising respiration. Though rare, it may be the clinical presentation of esophageal perforation, especially if it is unilateral and right-sided. Although esophageal perforation after a transesophageal echocardiography (TEE) is a known complication, misdiagnosis remains to be the most important contributing factor in the continuing morbidity and mortality rate.

## Case Description

**Figure 1.**
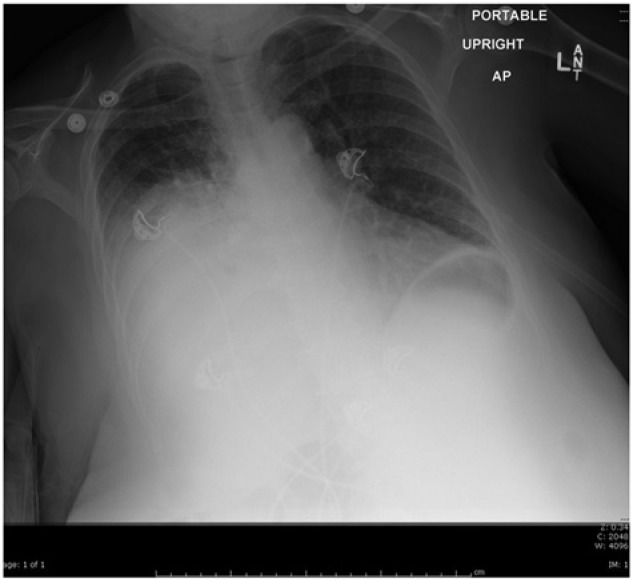
Chest X-ray presenting opacity in the right hemithorax.

We are presenting the case of a 61-year-old African American female patient who was admitted to the intensive care unit with right-sided weakness, numbness, and slurred speech. Further workup revealed a left cortical and cerebellar hemisphere ischemic stroke. TEE was performed as part of stroke management, which was uneventful. Feeding and scheduled oral medicines were started via a Dobbhoff tube, and further stroke management was optimized. On the third hospital day, she developed acute respiratory distress, warranting endotracheal intubation. Chest radiograph revealed a near complete opacification of right hemithorax with slight mediastinal shift (Figure 1). Preliminary diagnosis was aspiration pneumonia. Treatment with broad-spectrum antibiotics was started after cultures were obtained. Bronchoscopy showed copious, thick, and purulent secretions in the right middle and lower lobar area. However, quantitative culture of bronchoalveolar lavage showed no bacterial growth.

**Figure 2.**
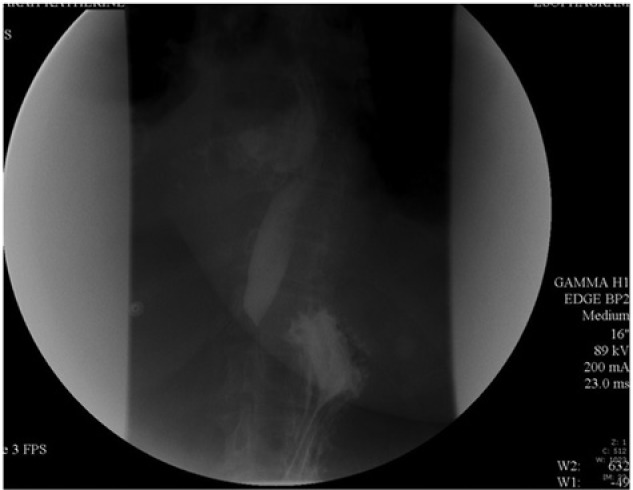
Leak of barium contrast from esophagus.

The pleural effusion persisted despite thoracentesis. Samples obtained were consistent with exudative effusion (low amylase, high lactate dehydrogenase, low glucose). A chest tube was inserted and drained a fair amount of purulent-appearing fluid. Overtime, her chest tube drainage slowed down but diminished-but-persistent effusion was still obvious in the chest X-ray. Due to notable clinical improvement in her pulmonary status, the patient was weaned off the mechanical ventilatory support and extubated after the 14th day of intubation. Per protocol, she was then referred to the speech therapist for a swallow assessment. A fluoroscopic dynamic swallow assessment showed a clear leaking from the esophagus. A chest computed tomography scan and esophagogram confirmed the leakage of contrast from distal esophagus (esophageal perforation) into the patient’s right chest cavity (Figure 2). There was no evidence of mass lesion noted.

The patient underwent primary repair of the perforated esophagus with muscle flap construction. Preoperative esophagoscopy demonstrated an esophageal tear with no distal obstruction or masses. Intraoperatively, a 30-mm perforation at the distal esophagus with marked contamination of right pleural space was noted but no evidence of mediastinitis was noted. Suspicious pleural lesions were sent to Pathology and were consistent with non–small cell carcinoma. The patient had a protracted recovery but was eventually discharged home.

## Discussion

Approximately 1.5 million pleural effusions are diagnosed in the United States each year.^1^ Pleural effusion represents an abnormal collection of fluid in the pleural space brought about by physiologic alteration in its absorption and production.^2^ It is an indicator of an underlying disease process that may be pulmonary or nonpulmonary in origin and may be acute or chronic. Although the etiological spectrum of pleural effusion is extensive, most pleural effusions are caused by congestive heart failure, pneumonia, malignancy, or pulmonary embolism.

Classification of the type of pleural effusion is based on the mechanism of fluid formation and biochemical assessment of the fluid. It can either be transudates, resulting from an imbalance between hydrostatic and oncotic pressures, or exudates, which results from inflammation of the pleura or decreased lymphatic drainage. In some cases, pleural fluids may present with a combination of both characteristics.^2^

This patient presented with an isolated right-sided pleural effusion, which was thought to be due to pneumonia and atelectasis. Etiology remained elusive until postextubation, wherein she had a swallow study that revealed esophageal perforation. The presentation of esophageal perforation has been described extensively in the literature. With technological advancement, incidences of esophageal perforation have increased due to easier recognition of such serious problems, and the etiology has changed from mostly spontaneous or traumatic to mostly iatrogenic.^3^ The frequency of esophageal perforation is 3 in 100,000 in the United States.^4^ The most common cause of esophageal perforation is medical instrumentation for diagnostic and therapeutic endeavors; in one series, such instrumentation caused 65% of all perforations.^5^ Within the instrumentation-related iatrogenic esophageal perforation, we would like to focus on perforation attributed to TEE.

TEE is one of the most valuable diagnostic tools that helps rapidly assess cardiac functionality. However, as in all procedures, there are some risks involved with this technique.^6^ Esophageal perforation after TEE is extremely low. A documented complication rate of 0.18% and a mortality rate of 0.98% were reported in the literature following TEE procedures involving 10,419 patients.^7^

Diagnosis of iatrogenic esophageal perforation following TEE is difficult and a high index of suspicion is necessary. The clinical features of esophageal perforation varied in terms of the location, cause, and interval between the time of injury to diagnosis. Our patient developed respiratory distress 3 days after the procedure and was subsequently intubated. This situation made it more difficult to diagnose since the septic picture may have clouded the underlying cause. The presentation is generally nonspecific and may even mimic disorders such as myocardial infarction, peptic ulcer perforation, pancreatitis, aortic aneurysm dissection, spontaneous pneumothorax, or pneumonia.^3^ Our patient’s clinical picture is consistent with the one described by MacGowan in a ventilated patient wherein uncontrolled mediastinal and pulmonary sepsis was the only diagnostic clue.^8^ Anatomically, the esophagus lacks a serosal layer and hence is more prone to rupture. It has been found that the location at greatest risk of instrumental injury is the Killian’s triangle, which is formed by the inferior constrictor pharyngeus and the cricopharyngeus muscles. In this region, the posterior esophageal mucosa is unprotected by muscularis layer and is separated from the retroesophageal space by buccopharyngeal fascia only.^9^ Once perforation developed, gastric contents, saliva, and other substances may enter the mediastinum and present as mediastinitis. A polymicrobial invasion of bacteria can also present as sepsis.^10^ The purported mechanism for pleural effusion can be explained by the inherent negative intrathoracic pressure causing the gastric fluid to be drawn in if the pleura has been violated. Another theory is that, if there is no evidence of pleural violation, a sympathetic pleural effusion often occurs. The effusion is usually left-sided, but can be bilateral.^11,12^ Though rare, an isolated right-sided pleural effusion can occur similar to what happened in our patient.^13^

Diagnosis of esophageal perforation can still be an enigma. A thorough history and physical examination with close attention to detail is essential. A simple chest X-ray is inconclusive, especially if the effusion is unilateral.^14^ Findings of pneumothorax, pneumopericardium, pleural effusion, and subdiaphragmatic air suggest esophageal perforation.^15^ Clearly, contrast esophagography remains to be the gold standard diagnostic tool. Which contrast to use is still controversial and dependent on the physician’s preference.^15-17^ A “positive” contrast study provides the level of perforation and presence or absence of extension into the pleural cavity. A “negative” study warrants either CT or direct visualization with flexible esophagoscopy. Abnormal findings suggestive of esophageal perforation include extraluminal air in the soft tissues of the mediastinum, esophageal thickening, perceptible communication of the air-filled esophagus with a contiguous mediastinal or paramediastinal air-fluid collection, or abscess cavities adjacent to the esophagus in the pleural space or mediastinum.^3,18^ Our patient had documented pleural effusion as shown on the chest X-ray and, not until after the swallow study, had the esophageal perforation been found. The findings on CT further confirmed the diagnosis.

The temporal factor from initiation of treatment determines the outcome after esophageal perforation. Our patient was given antibiotic therapy, effusion was continuously drained, and surgical repair of esophagus was performed as soon as the definitive diagnosis of perforation was confirmed. Current literature suggests that a delay in treatment for greater than 24 hours is associated with increased morbidity and mortality.^19^ The tenet has been supported by Brinster that mortality is increased if diagnosis and treatment was delayed more than 24 hours.^3^ However, recent literature has postulated that the underlying conditions, that is, pulmonary comorbidities, presence of sepsis, and mechanical ventilation are associated with significant increase in the risk of mortality in addition to the wait time for treatment.^3,20^ Vital treatment besides repair of perforation includes elimination of infection through initiation of systemic antibiotic therapy, nutritional support, and prevention of further contamination from the perforation.^21^ Successful treatment requires awareness and early intervention. Treatment plans involve conservative and operative management approaches.^22^ The decision on how to approach such a problem is influenced by the patient’s underlying comorbidities such as sepsis, shock, and respiratory failure. Regardless, all cases should warrant surgical consultation since it is imperative that surgical correction is the definitive treatment.

The prognosis in pleural effusion varies in accordance with the condition’s underlying etiology. Following TEE, if sepsis and acute respiratory distress develops, it is essential to be aware of potential complications of such a procedure. Additionally, one also needs to recognize that an expected complication appearing in an unexpected format and placement should result in further suspicion of causal mechanisms. Failure to recognize such injury such as an esophageal perforation may bring fatal outcomes.

In conclusion, purulent-appearing pleural effusions are not always due to infection. In spite of extremely low incidence, esophageal perforation needs to be considered as a rare complication of TEE. Although esophageal perforation typically presents as a left-sided pleural effusion, a right-sided pleural effusion can sometimes be the initial presentation and must be included in the differential diagnosis. Finally, swallow assessments are helpful components of care in otherwise improving critically ill patients.
